# The impact of 4-week high-intensity interval training on mental health and sleep quality in female college students with normal weight obesity: a randomized controlled trial

**DOI:** 10.1186/s12967-025-07276-7

**Published:** 2025-11-06

**Authors:** Jiawen Fu, Wangyi Zhang, Xinyi Xu, Xinyi Mao, Liyan Wang, Ming Cai, Ruoyu Yang

**Affiliations:** College of Rehabilitation Sciences, Shanghai University of Medicine and Health Sciences, Shanghai, China

**Keywords:** Normal weight obesity, High-intensity interval training, Depressive state, Anxiety state, Sleep quality

## Abstract

**Background:**

Normal Weight obesity, characterized by normal-range body mass index accompanied by elevated adiposity, poses a potential health challenge among female college students. This demographic exhibits heightened susceptibility to psychological disturbances and sleep disorders. High-Intensity Interval Training (HIIT) appears to have potential in managing metabolic dysregulation. However, its efficacy in improving mental health and sleep quality in individuals with normal weight obesity remains uncertain.

**Methods:**

This randomized controlled trial enrolled female college students (*N* = 35) who met the criteria for normal weight obesity, defined as a body mass index between 20 and 23 kg/m² and a body fat percentage greater than 30%. The intervention group (*n* = 17) participated in a 4-week HIIT protocol, consisting of five sessions per week at greater than 90% VO_2_max, while the control group (*n* = 18) received standard health education. Validated instruments were utilized to assess depression (PHQ-9), anxiety (GAD-7), and sleep quality (PSQI). Trial registration: ChiCTR2100050711 Registered 3 September 2021 Retrospectively registered, https//www.chictr.org.cn/showproj.html? proj=132,914.

**Results:**

The HIIT intervention significantly reduced PHQ-9 scores from 5.8 ± 4.2 to 3.2 ± 2.7 (*p* < 0.01) and GAD-7 scores from 5.1 ± 5.3 to 2.8 ± 2.9 (*p* < 0.05), while also improving PSQI scores from 5.1 ± 3.4 to 3.5 ± 2.5 (*p* < 0.01). Post-intervention analyses revealed a strong correlation between sleep quality and levels of depression (*R* = 0.813, *p* < 0.001) and anxiety (*R* = 0.739, *p* < 0.001) in the HIIT group, whereas no significant change in sleep quality was observed among the control group.

**Conclusion:**

This study demonstrates the therapeutic efficacy of HIIT in improving psychological disturbances and sleep architecture in female students with normal weight obesity. It may serve as a time-efficient intervention. The observed correlation between sleep quality and psychological parameters warrants further investigation into the underlying mechanisms.

## Introduction

Obesity has emerged as a significant public health concern worldwide, influencing both physical and mental health [1–3]. Among the various forms of obesity, normal weight obesity (NWO), characterized by an elevated body fat percentage despite a normal body weight, is particularly prevalent among female college students [4, 5]. This demographic is characterized by reduced skeletal muscle mass, decreased physical fitness, and elevated cardiometabolic risk factors, coupled with a tendency towards sedentary behavior, which may contribute to associated health issues [5, 6]. Studies have reported a significant correlation between obesity and psychological issues, such as depression and anxiety, among adolescents and young adults, with obesity significantly increasing the risk of developing these mental health problems [7, 8]. As a specific form of obesity, normal weight obesity may also adversely affect the psychological well-being of this population, a relationship preliminarily confirmed in the pilot experiment conducted for subject screening in this study. Furthermore, the interaction between mental health and obesity creates a challenging cycle, where psychological distress leads to unhealthy coping mechanisms, such as sedentary behavior and emotional eating [9, 10].

Female college students frequently report experiencing sleep disturbances, which further complicates the relationship between obesity and mental health [11, 12]. Poor sleep quality can impair cognitive function, reduce motivation for physical activity, and increase the risk of depressive and anxiety disorders [13, 14]. Similar cases have been frequently reported in obese populations, while reports on normal weight obesity are scarce [15, 16]. However, our preliminary screening experiments have demonstrated differences in sleep quality between individuals with normal weight obesity and those with normal body weight and fat percentage. This finding suggests that sleep quality issues may also be present among female college students with normal weight obesity, potentially indicating an association with mental health that may necessitate intervention.

Recent studies have increasingly highlighted the positive impact of physical activity on mental health [17, 18]. Regular exercise is associated with reduced depressive symptoms and anxiety levels. Additionally, it can improve the overall mood [19, 20]. High-Intensity Interval Training (HIIT) has gained particular attention for its efficiency in enhancing cardiovascular fitness and reducing body fat in a relatively short time [21, 22]. HIIT involves alternating short bursts of high-intensity exercise with recovery periods or lower-intensity activities [23], appealing to young people who are often busy and have limited time for traditional exercise.

Despite the promising benefits of HIIT, its effects on psychological well-being and sleep quality, particularly among female college students with normal weight obesity, have not been extensively studied. This gap in the literature underscores the importance of the current study, which aims to investigate the effects of a 4-week HIIT intervention on depression, anxiety, and sleep quality in this vulnerable group. Using a randomized controlled trial design, we aim to evaluate the efficacy of HIIT not only in enhancing physical fitness but also in providing significant psychological benefits. The anticipated outcomes of the study are twofold. First, we expect improvements in the mental health indicators of depression and anxiety among participants engaged in the HIIT regimen. Second, we seek to evaluate the relationship between these improvements and sleep quality. Previous research suggests a nuanced interplay between exercise, mental health outcomes, and sleep, indicating that enhanced mental health can lead to better sleep patterns, while poor sleep can exacerbate mental health issues. Furthermore, the findings from this study are expected to inform future interventions aimed at improving both physical and mental health among female college students with normal weight obesity. When exercise is incorporated into mental health treatment plans, healthcare providers can better address the complex needs of this population, ultimately enhancing their quality of life. These effects may not only impact individual health but also contribute to healthier college environments and improve academic performance. To sum up, this study aims to illuminate the multifaceted relationship between obesity, mental health, and sleep, offering evidence-based insights to inform effective health promotion strategies. By focusing on an innovative exercise approach like HIIT, we hope to empower female college students to improve their health both physically and mentally, thereby helping them break the cycle of obesity and psychological distress.

## Materials and methods

### Ethics approval

This study was conducted in compliance with the Declaration of Helsinki and was approved by the Ethics Committee of Shanghai University of Medicine and Health Sciences, China. Written informed consent was obtained from all participants.

### Study design

**Fig. 1:**
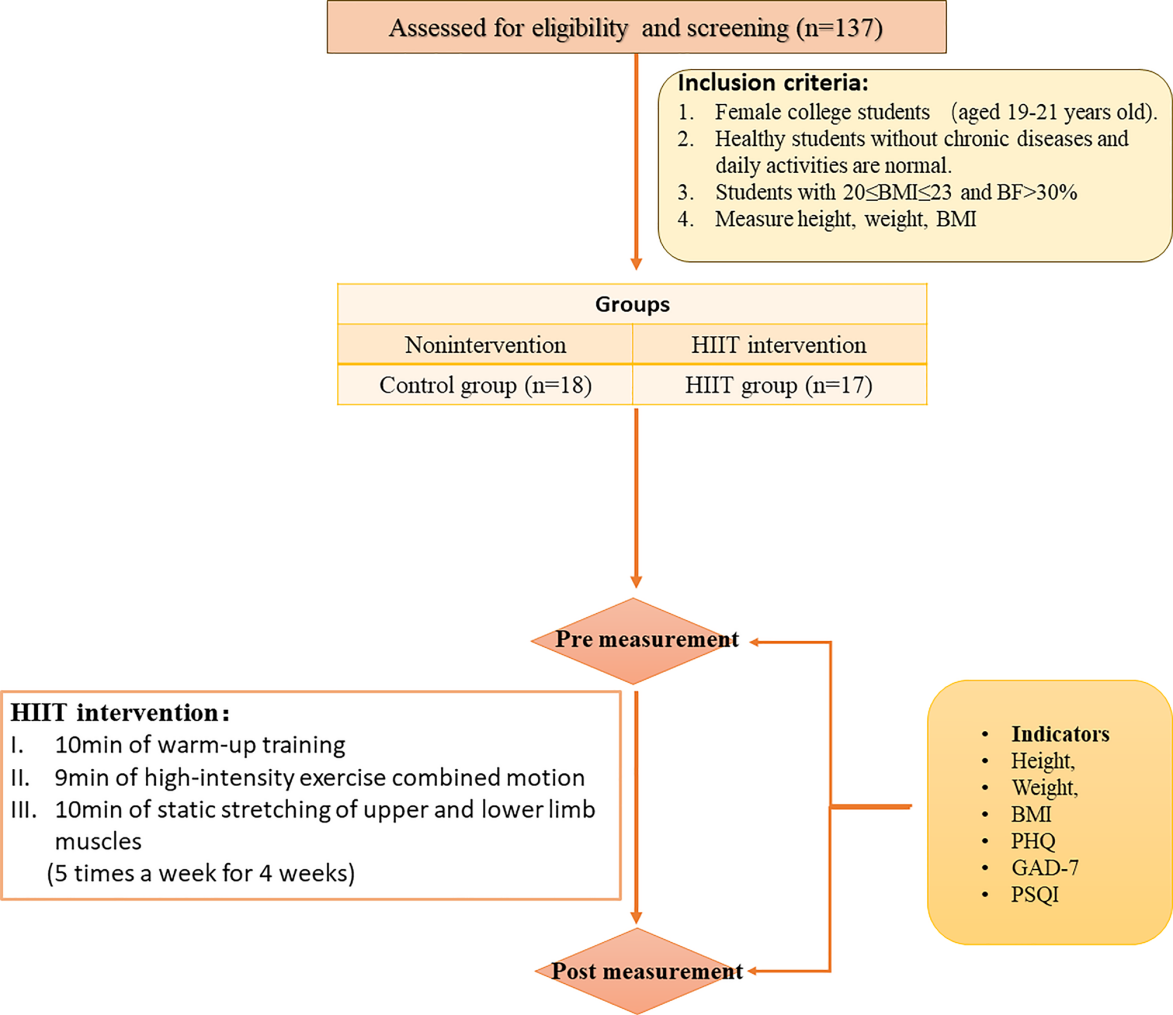
Flowchart of study design

The study design involved screening female college students at Shanghai University of Medicine and Health Sciences for normal weight obesity. Eligible participants were randomly selected and assigned to either the HIIT group or the control group. Using a randomized controlled trial method, the HIIT group participated in a 4-week high-intensity interval training program, while the control group received no interventions during the study period. Assessments of body composition, mental health, and sleep quality were conducted before and after the 4-week intervention to evaluate the impact of high-intensity interval training. Data from these assessments were analyzed to determine the effectiveness of the training program. A detailed study design is available in Fig. 1.

### Participants

A cohort of 137 female volunteers was recruited from Shanghai University of Medicine and Health Sciences. Inclusion required meeting the criteria for normal weight obesity as defined by the Japan Obesity Society [24], which includes two key parameters: (1) a body mass index (BMI) within the normal weight range (BMI = weight/height², where 20 ≤ BMI ≤ 23, with weight in kilograms and height in meters), and (2) a body fat percentage exceeding 30%. This dual-criterion approach enables the identification of individuals exhibiting the paradoxical phenotype of normal weight obesity, characterized by a normal BMI concurrent with elevated adiposity.

Using results from the InBody 770 body composition analyzer, 20 participants were randomly selected as subjects with normal weight obesity; ultimately, 17 completed the training intervention (mean age: 19.2 ± 1.1 years; height: 164.5 ± 4.8 cm; weight: 59.1 ± 3.5 kg; BMI: 21.8 ± 0.9 kg/m²; body fat percentage: 33.5 ± 2.3%). The control group consisted of 18 female college students who also met the criteria for normal weight obesity and completed both pre- and post-intervention assessments (mean age: 19.3 ± 1.3 years; height: 162.4 ± 5.4 cm; weight: 58.2 ± 6.3 kg; BMI: 22.1 ± 2.0 kg/m²; body fat percentage: 32.2 ± 2.6%). Prior to the study, these participants received only health education and did not engage in any training. All participants had no history of cardiovascular disease, endocrine disorders, or other contraindications to exercise. Relevant assessments were conducted before and after the training intervention for the experimental group. Baseline characteristics of participants are presented in Table 1.

**Table 1:** The baseline characteristics of participants

		Control (*n* = 18)	HIIT (*n* = 17)	*p*-value
Age (y)		19.3 ± 1.3	19.2 ± 1.1	0.857
Height (cm)		162.4 ± 5.4	164.5 ± 4.8	0.205
Body mass (kg)		58.2 ± 6.3	59.1 ± 3.5	0.588
BMI (kg/m^2^)		22.1 ± 2.0	21.8 ± 1.0	0.606
Body fat percentage (%)		32.2 ± 2.6	33.5 ± 2.3	0.424

### HIIT intervention protocol

The subjects underwent a one-week adaptive training program intended to familiarize them with the course, learn proper movements, and gradually adapt to the training intensity. Following this period, they commenced a 4-week training program with a frequency of five sessions per week. The training intensity was maintained at over 90% of the maximum oxygen uptake. Prior to each session, participants engaged in a ten-minute warm-up to prevent injury. This was followed by a nine-minute period of high-intensity exercises. Finally, a five-minute breathing relaxation exercise was conducted, and static stretching of the upper and lower limb muscles was performed for ten minutes.

### Training programs

The ten-minute warm-up exercise consisted of hand exercises, flexibility training, five minutes of muscle stretching, and five minutes of joint activity.

The nine-minute high-intensity exercise routine included opening and closing jumps, squats, push-ups, supine and bent-knee raises, step jumps, abdominal rolls, hip bridges, poppy jumps, and lunge squats. Each exercise lasted for 40 s, followed by a 20-second interval.

The inter-group rest period was one minute, with a total of three exercise sets. The entire HIIT process was supervised by professional coaches, and during testing, trained personnel monitored the physical condition of the participants in real time. In the event of any sports-related injuries or fatigue following the testing, the sports coach provided supervision and guidance.

### Measurements

#### Height and body composition

Participants were provided with detailed instructions regarding the body composition analysis procedure and were advised to avoid intense physical activity for at least eight hours prior to the measurement. Height was measured using a calibrated stadiometer, while body composition (body mass and body fat percentage) was assessed using the InBody-770 bioelectrical impedance analysis (BIA) device (Korea). Body mass index (BMI) values were calculated using the recorded height and body mass measurements.

#### Depression

The Patient Health Questionnaire-9 (PHQ-9) is a widely used depression screening tool consisting of nine items that assess various symptoms of depression. Each item provides four response options corresponding to different frequencies of symptom occurrence, allowing respondents to select the option that best represents their situation. To effectively assess changes in the severity of depressive symptoms, the PHQ-9 focuses on symptoms experienced over the past two weeks. Each question is scored from 0 to 3 points, reflecting the frequency of the reported symptoms. The total score is obtained by summing the scores across all nine items, resulting in a range from 0 to 27 points. The interpretation of the total score is as follows: 0–4 points indicate the absence of depressive symptoms; 5–9 points suggest mild depressive tendencies; 10–13 points indicate moderate depressive tendencies; 14–18 points reflect moderate to severe depressive tendencies; and 19–27 points signify a tendency towards severe depression [25].

#### Anxiety

The Generalized Anxiety Disorder 7-item scale (GAD-7) is an established screening tool designed to assess anxiety symptoms through seven questions, each corresponding to different types of anxiety or potential symptoms. Respondents are provided with four answer options for each question, reflecting varying levels of anxiety symptom severity, and are instructed to select the response that best describes their current situation. To effectively evaluate changes in the severity of anxiety symptoms, the GAD-7 specifically focuses on symptoms experienced within the past two weeks. Each item is scored from 0 to 3 points, based on the frequency of the symptoms as outlined in the question. The cumulative score from all seven items yields a total score ranging from 0 to 21 points. The interpretation of the total score is categorized as follows: 0–4 points indicate no anxiety symptoms; 5–9 points suggest a mild tendency toward anxiety; 10–13 points reflect a moderate tendency; 14–18 points indicate moderate to severe anxiety tendencies; and 19–21 points signify a severe tendency toward anxiety [26].

#### Sleep quality

The Pittsburgh Sleep Quality Index (PSQI) consists of 19 self-rated questions, supplemented by five questions assessed by a bed partner or roommate. For scoring purposes, the focus is primarily on the initial 19 items. These items are categorized into seven distinct dimensions vital for understanding sleep quality: sleep onset latency, sleep duration, sleep efficiency, subjective sleep quality, sleep disturbances, daytime dysfunction, and the use of hypnotic medications. Each question is scored from 0 to 3, reflecting increasing symptom severity. The total score for each dimension is calculated using various independent methods, with the cumulative score from all seven dimensions representing the overall sleep quality score. The total score ranges from 0 to 21 points, where higher scores indicate poorer sleep quality. Specifically, the interpretation of the total score is as follows: 0–5 points indicate good sleep quality; 6–10 points suggest decent sleep quality; 11–15 points reflect average sleep quality; and 16–21 points signify poor sleep quality [27].

### Data analysis

Descriptive statistics were reported as mean ± standard deviation. Independent samples t-tests were used to assess differences between groups for normally distributed data, while paired t-tests were employed for within-group comparisons (pre-intervention and post-intervention). For repeated measures, analysis of variance (ANOVA) was performed, along with correlation analyses involving three variables. All data analyses were conducted using SPSS version 26.0 and R language version 4.2.1. Significance levels were set at *p* < 0.05 and *p* < 0.01.

## Results

Our previous study has reported significant reductions in BMI and body fat percentage among female college students with normal weight obesity following a 4-week high-intensity interval training (HIIT) intervention. Specifically, both BMI and body fat percentage decreased significantly post-intervention compared to pre-intervention measurements, with the reduction in body fat percentage being particularly notable (*p* < 0.05). These results suggest that the 4-week HIIT program has a positive impact on the body composition of female students with normal weight obesity [28]. This observation prompts an investigation into whether the 4-week HIIT intervention also influences mental health indicators and sleep quality in these individuals. The results section below will detail the effects of the 4-week HIIT intervention on mental health and sleep quality in female college students with normal weight obesity.

### Effects of HIIT on depressive state

**Fig. 2:**
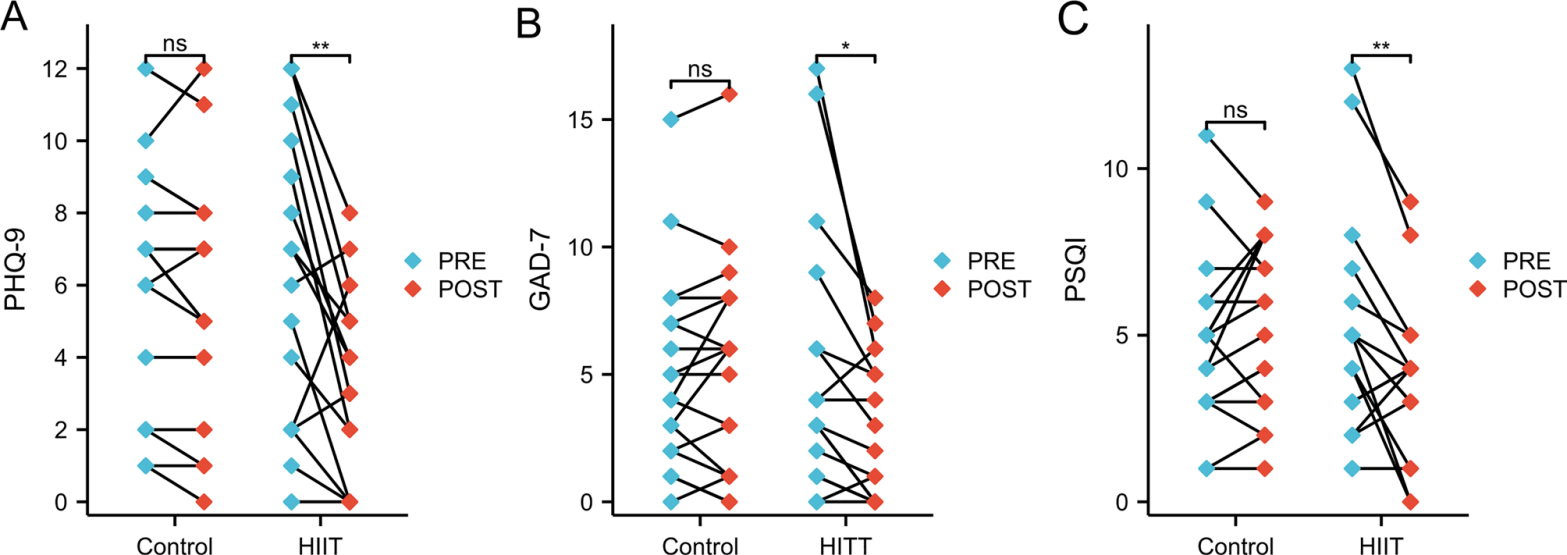
The comparison results of depressive state, anxiety state, and sleep quality between pre- and post- HIIT intervention in the Control and HIIT groups. (**A**): The comparison of PHQ-9 scores between pre- and post- intervention in the Control and HIIT groups; (**B**): The comparison of GAD-7 scores between pre- and post- intervention in the Control and HIIT groups; (**C**): The comparison of PSQI scores between pre- and post- intervention in the Control and HIIT groups

Regarding depressive states, the control group exhibited no significant changes in PHQ-9 scores, which were 6.2 ± 3.6 before the intervention and 5.8 ± 3.6 after four weeks (*p* > 0.05). In contrast, the HIIT group showed a significant reduction in PHQ-9 scores, which decreased from 5.8 ± 4.2 before the HIIT intervention to 3.2 ± 2.7 afterward (*p* < 0.01) (Fig. 2A). The two-way mixed ANOVA revealed a significant time×group interaction effect (F = 7.180, *p* = 0.011; F_Adj._=9.114, *p*_Adj._=0.005). Further analysis of single effects indicated no significant in pre-intervention measurements (F = 0.123, η²=0.004, *p* = 0.728), but a significant difference in post-intervention measurements (F = 5.483, η²=0.142, *p* = 0.025). These findings suggest that the 4-weekHIIT protocol elicited statistically significant reductions in depressive states among female college students with normal weight obesity. Detailed results are presented in Table 2.

**Table 2:** Effects of HIIT on mental health and sleep quality

	Control (*n* = 18)		HIIT (*n* = 17)		F (Two-way mixed ANOVA)	*p*-value	F (Two-way mixed ANOVA)Adj.	*p*-value Adj.	F (Simple effect)		*p*-value		η2	
	Pre	Post	Pre	Post					Pre	Post	Pre	Post	Pre	Post
PHQ-9	6.2 ± 3.6	5.8 ± 3.6	5.8 ± 4.2	3.2 ± 2.7	7.180	**0.011**	9.114	**0.005**	0.123	5.483	0.728	**0.025**	0.004	0.142
GAD-7	5.1 ± 3.8	5.4 ± 4.2	5.1 ± 5.3	2.8 ± 2.9	8.333	**0.007**	6.751	**0.014**	0.001	4.449	0.973	**0.043**	3.431E-05	0.119
PSQI	4.8 ± 2.5	5.5 ± 2.5	5.1 ± 3.4	3.5 ± 2.5	11.692	**0.002**	8.277	**0.007**	0.077	5.639	0.783	**0.024**	0.002	0.146

Repeated measures ANOVA followed by simple effect test analysis were used

Data were presented as mean ± SE

Adj.: Correct repeated measures ANOVA by adjusting for confounding factors such as Age, BMI, and Body fat percentage in Table 1

*p* < 0.05 marked in bold

### Effects of HIIT on anxiety state

The control group showed no significant difference in the GAD-7 scores, which were 5.1 ± 3.8 before the intervention and 5.4 ± 4.2 after four weeks (*p* > 0.05). Conversely, the HIIT group experienced a significant reduction in GAD-7 scores, decreasing from 5.1 ± 5.3 before the HIIT intervention to 2.8 ± 2.9 afterward (*p* < 0.05) (Fig. 2B). The two-way mixed ANOVA revealed a significant time×group interaction effect (F = 8.333, *p* = 0.007; F_Adj._=6.751, *p*_Adj._=0.014). Further analysis of single effects showed no significant in pre-intervention measurements (F = 0.001, η²=3.431E-05, *p* = 0.973), but a significant difference in post-intervention measurements (F = 4.449, η²=0.119, *p* = 0.043). These findings indicate that the 4-week HIIT protocol also elicited statistically significant reductions in anxiety levels among female college students with normal weight obesity. Detailed results are presented in Table 2.

### Effects of HIIT on sleep quality

Regarding sleep quality, the control group exhibited no significant change in PSQI scores between pre- and post-intervention measurements (4.8 ± 2.5 vs. 5.5 ± 2.5, *p* > 0.05) over the 4-week period. The HIIT group demonstrated a significant improvement in PSQI scores from baseline to post-intervention (5.1 ± 3.4 vs. 3.5 ± 2.5, *p* < 0.01), with scores decreasing notably after the intervention (Fig. 2C). Two-way mixed ANOVA analysis of the time×group interaction revealed a significant effect (F = 11.692, *p* = 0.002; F_Adj._=8.277, *p*_Adj._=0.007). Examination of single effects showed no significant difference at baseline (F = 0.077, η^2^ = 0.002, *p* = 0.783), while post-intervention measurements indicated a significant difference (F = 5.639, η^2^ = 0.146, *p* = 0.024). These findings suggest that the 4-week HIIT exercise intervention significantly improved sleep quality in female college students with normal weight obesity. Detailed results are presented in Table 2.

### Correlation analysis between sleep quality and mental health

**Fig. 3:**
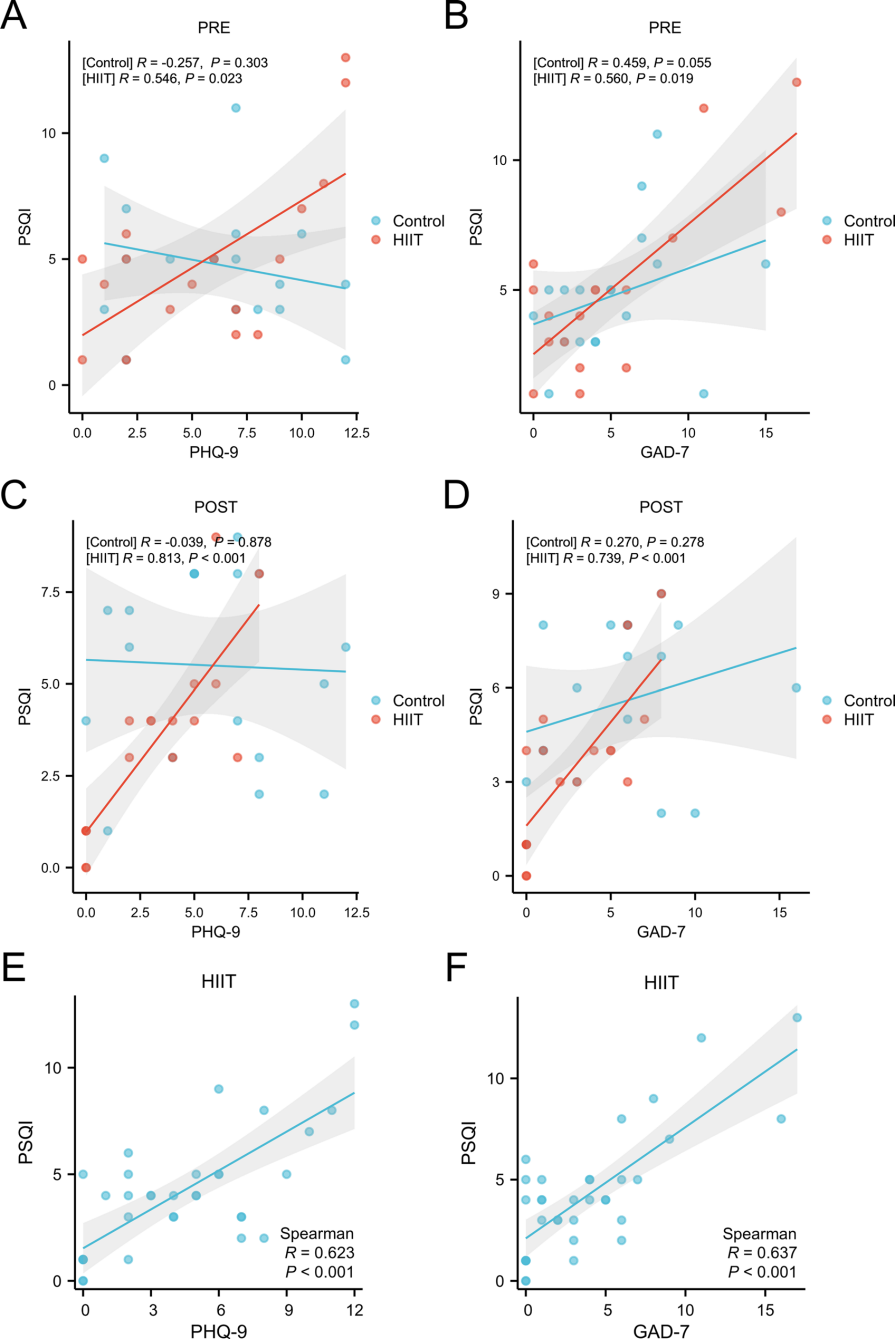
The correlation analysis between sleep quality and mental health in the Control and HIIT groups. (**A**): The correlation between PSQI scores and PHQ-9 scores before intervention in the Control and HIIT groups; (**B**): The correlation between PSQI scores and GAD-7 scores before intervention in the Control and HIIT groups; (**C**): The correlation between PSQI scores and PHQ-9 scores after intervention in the Control and HIIT groups; (**D**): The correlation between PSQI scores and GAD-7 scores after intervention in the Control and HIIT groups; (**E**): The correlation between PSQI scores and PHQ-9 scores before and after intervention of the two groups; (**F**): The correlation between PSQI scores and GAD-7 scores before and after intervention of the two groups

Correlation analysis revealed no significant relationships between sleep quality (PSQI scores) and depression (PHQ-9 scores) or anxiety (GAD-7 scores) in the control group at both pre- and post-intervention measurements (*p* > 0.05) (Fig. 3A and D). However, in the HIIT group, significant positive correlations between sleep quality and mental health indicators were observed. At baseline, PSQI scores were significantly positively correlated with both PHQ-9 scores (*R* = 0.546, *p* < 0.05) (Fig. 3A) and GAD-7 scores (*R* = 0.560, *p* < 0.05) (Fig. 3B). These correlations strengthened at post-intervention, with PSQI scores demonstrating higher positive correlations with PHQ-9 scores (*R* = 0.813, *p* < 0.001) (Fig. 3C) and GAD-7 scores (*R* = 0.739, *p* < 0.001) (Fig. 3D). Further correlation analysis combining both pre- and post-intervention data for the HIIT group revealed that sleep quality maintained significant positive correlations with depression (*R* = 0.623, *p* < 0.001) (Fig. 3E) and anxiety states (*R* = 0.637, *p* < 0.001) (Fig. 3F). These findings suggest that throughout the HIIT intervention period, sleep quality exhibited strong positive correlations with both depression and anxiety levels.

## Discussion

Prior research has established complex relationships between obesity and various psychological and physiological parameters [29, 30]. Regarding sleep quality, several studies have indicated that individuals with a high body fat percentage often experience poor sleep quality [31, 32]. However, these studies typically focus on the association between elevated body fat percentage and poor sleep quality, rarely specifying whether participants have normal BMI levels. Sleep quality is a multifactorial outcome potentially influenced by various factors, including body fat percentage. Although our findings demonstrate that a HIIT intervention can improve sleep quality in female college students with high body fat percentage and normal weight, caution is warranted in asserting that this demographic inherently experiences poorer sleep quality due to the limited supporting literature.

The relationship between body fat percentage and depression has been documented in previous studies [33, 34]. Wang et al. found that the increased risk of obesity-related depression appears to partially depend on metabolic health status, suggesting that even non-obese populations should be screened for metabolic disorders [35]. This finding indicates that individuals with a high body fat percentage may be at increased risk for obesity-related depression. Our results are generally consistent with this finding and further demonstrate that a HIIT intervention can effectively improve depressive symptoms in female college students.

We observed strong positive correlations between sleep quality and psychological parameters, specifically depression and anxiety, in the HIIT group, particularly post-intervention. Previous studies have reported moderate correlations between sleep quality and psychological parameters in college student populations (*R* = 0.3–0.5) [36, 37]. However, our study revealed stronger correlations (*R* > 0.7) specifically in individuals with normal weight obesity after HIIT intervention. This suggests that the relationship between sleep and psychological well-being may be particularly sensitive to exercise intervention in this population. It is also worth noting that our study found no significant correlations in the control group, which contrasts with the strong correlations observed in the HIIT group and differs from previous research that typically reported baseline correlations regardless of intervention status. This unique observation suggests that HIIT may function as a catalyst, enhancing the relationship between sleep quality and psychological health in individuals with normal weight obesity. This phenomenon has not been previously documented in the literature. Collectively, these findings enhance our understanding of the complex interrelationships among normal weight obesity, sleep quality, and psychological well-being, suggesting that HIIT is a promising intervention strategy for this specific population. Future research should explore the underlying mechanisms mediating these relationships and the long-term sustainability of these improvements.

Normal weight obesity is characterized by an elevated body fat percentage, and emerging research suggests this form of obesity warrants serious clinical attention [38]. The diagnostic criteria emphasize body fat percentage over the conventional BMI metric, highlighting that adiposity, rather than weight alone, is the primary determinant of obesity-related health risks [39]. Visceral adipose tissue serves as a principal site for producing pro-inflammatory cytokines [40], which may affect sleep homeostasis. Elevated concentrations of these circulating cytokines can impair sleep architecture and efficiency. Initially, monogenic normal weight obesity phenotypes were presumed to be associated with increased visceral adiposity. However, subsequent analysis suggested this observation could explain a potential mechanistic pathway linking normal weight obesity to sleep dysregulation [41]. This relationship manifests as an intricate bidirectional feedback loop: sleep disturbances can enhance activity within reward-associated neural circuits, potentially increasing preferences for energy-dense foods and consequent hyperphagia, thereby perpetuating adipose tissue expansion. The neurobiological connections between normal weight obesity and psychological disturbances, particularly depression and anxiety, remain incompletely understood. One proposed mechanism involves dysregulation of the hypothalamic-pituitary-adrenal (HPA) axis, wherein altered cortisol secretion patterns may mediate the relationship between adiposity and psychiatric manifestations [42]. Additionally, neural alterations associated with obesity resemble those seen in depression, including region-specific changes in cellular density, compromised neural connectivity, and altered neuronal excitability [43]. Our findings of significant correlations between sleep quality and mental health parameters, specifically depression and anxiety, align with previous research indicating that sleep deprivation can lead to depressive and anxiety-like behavioral phenotypes [44]. This convergence of evidence highlights the complex interplay between metabolic, sleep, and psychological parameters in individuals with normal weight obesity and underscores the importance of comprehensive therapeutic approaches addressing these multiple domains simultaneously. This synthesis of established findings and our novel observations provides a nuanced understanding of the complex relationships between normal weight obesity, sleep quality, and mental health. Our study observed strong correlations, particularly following HIIT intervention, suggesting potential therapeutic mechanisms worthy of further investigation.

The therapeutic efficacy of HIIT observed in our study may be attributed to multiple physiological and neurobiological mechanisms. HIIT exerts significant modulatory effects on central neurotransmitter systems, enhancing the release of monoamines (dopamine, serotonin, and norepinephrine) and endogenous opioids, which are critical mediators for mood regulation and emotional balance [45]. Furthermore, HIIT has obvious effects on the secretion pattern of melatonin [46], which is believed to regulate circadian rhythm and improve sleep quality [47]. HIIT also promotes neuroplasticity and neurogenesis, particularly in the hippocampus, a region crucial for emotional regulation and stress response. This enhanced neural plasticity may underlie the observed improvements in depressive and anxiety symptoms [48]. Additionally, HIIT induces significant neuroendocrine effects by modulating the hypothalamic-pituitary-adrenal (HPA) axis, potentially reducing cortisol secretion and chronic stress responses. Its anti-inflammatory and antioxidant effects further contribute to its therapeutic efficacy. By reducing levels of pro-inflammatory cytokines (TNF-α, IL-1) and enhancing antioxidant enzyme activity, HIIT fosters a neuroprotective environment conducive to improved psychological well-being and sleep quality [49]. From a physiological standpoint, HIIT’s impact on cardiorespiratory fitness and body temperature regulation facilitates both sleep initiation and maintenance. The intermittent nature of HIIT elicits distinct thermogenic responses that may help optimize circadian entrainment [50]. These mechanistic insights provide a theoretical framework for understanding the observed improvements in depression, anxiety, and sleep quality within our study population, underscoring that HIIT’s therapeutic effects occur through multiple interconnected physiological and psychological pathways.

The aforementioned mechanisms provide a theoretical framework for understanding the observed phenomena. In terms of application, these mechanisms are particularly relevant to our study group, consisting of females aged around 19–20 years. This demographic group exhibits distinct physiological and psychological characteristics. Epidemiological evidence indicates that, compared to males, females have higher rates of anxiety and depression [51], which may influence their response to interventions like exercise. Additionally, this stage of development is marked by significant physiological and psychological changes, potentially making individuals more sensitive to exercise interventions. Due to it’s time-efficient and high-intensity nature, HIIT may be particularly beneficial for this group. The age- and gender- specific responses observed in our study underscore the importance of tailored intervention strategies in clinical practice.

Careful consideration of exercise parameters is crucial for interpreting our findings. The HIIT protocol employed in our study involved intensities exceeding 90% of maximal oxygen uptake and was administered five times weekly for four weeks. This regimen proved effective in enhancing both physical and psychological parameters. Previous research has demonstrated HIIT’s ability to rapidly improve cardiorespiratory fitness and optimize cardiovascular parameters [50]. The high-intensity nature of the intervention may facilitate psychological improvements through the up-regulation of brain-derived neurotrophic factor (BDNF), which may support neuroplasticity within affected neural circuits [52]. However, it is important to note that some studies have reported potential adverse effects of HIIT on the duration of rapid eye movement (REM) sleep, while non-REM sleep architecture remains unaffected [53]. These observations underscore the importance of optimizing exercise parameters-intensity, frequency, and duration-to maximize therapeutic outcomes while minimizing adverse effects on sleep architecture.

By comparing HIIT to other forms of exercise, we identified its unique advantages in cardiovascular fitness, fat reduction, and time efficiency [54]. Research has demonstrated that HIIT yields comparable outcomes to traditional aerobic exercise while requiring significantly less time. For example, one study compared HIIT with continuous training and found that HIIT provided sufficient aerobic stimulation while being far more time-efficient [55]. Additionally, another study reported that HIIT was more effective than traditional aerobic exercise in alleviating depressive symptoms [56]. Compared to Moderate-Intensity Continuous Training (MICT), HIIT has been shown to deliver similar or superior results in enhancing aerobic capacity, reducing body fat, and controlling body weight, while also being perceived as more enjoyable [57]. Furthermore, Racil et al. highlighted the superior health benefits of HIIT over MICT in obese adolescent females [58].

Several limitations should be considered when interpreting the current findings. First, the study exclusively involved female college students aged 19 to 20 with a specific type of obesity, potentially limiting the generalizability of the results to other populations, such as males or individuals of different ages and obesity types. Second, while the 4-week HIIT intervention showed significant improvements in psychological and sleep parameters, its short duration constrains conclusions regarding the long-term effectiveness and sustainability of these benefits. Sleep quality was assessed using the Pittsburgh Sleep Quality Index (PSQI), a validated tool that, however, only provides subjective measures. To enhance the evaluation of sleep quality, future studies should incorporate objective measures such as polysomnography or actigraphy. Similarly, the psychological parameters were evaluated using self-reported questionnaires (PHQ-9 and GAD-7), which may be susceptible to response bias. Additionally, although our study demonstrated strong correlations between sleep quality and psychological parameters, it did not directly investigate the underlying mechanisms of these relationships. Future research should include biochemical markers, such as inflammatory cytokines, BDNF, and stress hormones, to elucidate the physiological pathways through which HIIT exerts its beneficial effects. Finally, the study did not control for potential confounding factors such as dietary habits, academic stress levels, or menstrual cycle phases, which could influence both psychological state and sleep quality. Future research should address these factors to strengthen the evidence supporting HIIT as an effective therapeutic intervention for this population.

In conclusion, this study demonstrates that a 4-week high-intensity interval training (HIIT) intervention effectively improves sleep quality and reduces depression and anxiety symptoms among female college students with normal weight obesity. Notably, there are particularly strong connections observed between sleep quality and psychological parameters post-intervention. These findings enhance our understanding of the complex relationships between exercise, sleep, and mental health in populations affected by obesity and establish HIIT as a promising therapeutic option for this specific group. HIIT is time-efficient and offers numerous benefits, making it particularly appealing to college students who often have busy schedules and face various health challenges. Future research should aim to optimize HIIT protocols for diverse groups with normal weight obesity, using technology for precise monitoring and offering personalized interventions. The strong correlations observed between sleep and psychological parameters suggest potential therapeutic targets for combined interventions. Additionally, exploring the molecular and neurobiological mechanisms underlying these improvements could lead to more targeted and effective intervention methods. From a clinical perspective, these findings support integrating HIIT into comprehensive treatment programs for individuals with normal weight obesity, particularly those with concurrent sleep and psychological issues. The demonstrated efficacy of HIIT suggests its potential role in preventive healthcare strategies, especially in university settings where early intervention could significantly impact long-term health outcomes. As we continue to explore the complexities of normal weight obesity and its related complications, HIIT emerges as a valuable tool within the broader context of public health interventions for this increasingly prevalent condition.

## Data Availability

The datasets used and/or analyzed during the current study are available from the corresponding author on reasonable request.
